# Associations Between Food Insecurity, Psychological Distress and Disordered Eating Risk in University Students: Evidence from a Cross-Sectional Mediation Analysis

**DOI:** 10.3390/nu18111744

**Published:** 2026-05-29

**Authors:** Katherine Kent, Nina Glavincevski, Suvasish Das Shuvo, Clare E. Collins, Melinda Hutchesson, Karen E. Charlton

**Affiliations:** 1 School of Medical, Indigenous and Health Sciences, University of Wollongong, Wollongong, NSW 2500, Australia; katherinek@uow.edu.au (K.K.); ng977@uowmail.edu.au (N.G.); sds988@uowmail.edu.au (S.D.S.); 2 Department of Nutrition and Food Technology, Jashore University of Science and Technology, Jashore 7408, Bangladesh; 3 School of Health Sciences, College of Health, Medicine and Wellbeing, The University of Newcastle, Newcastle, NSW 2308, Australia; clare.collins@newcastle.edu.au (C.E.C.); melinda.hutchesson@newcastle.edu.au (M.H.); 4 Nutrition and Metabolic Health Research Program, Hunter Medical Research Institute, Newcastle, NSW 2305, Australia

**Keywords:** food insecurity, university students, higher education, Australia, psychological distress, disordered eating, mediation analysis

## Abstract

**Background/Objective:** Food insecurity is increasingly recognised as a concern among university students. Less is known about the interrelationships between food insecurity, psychological distress, and disordered eating risk in this population. This study aimed to examine associations between food insecurity, psychological distress, and disordered eating risk among university students, and to explore whether psychological distress mediates the association between food insecurity and disordered eating. **Methods:** A cross-sectional survey among university students assessed food insecurity using the USDA HFSSM. Psychological distress was assessed using the Kessler Psychological Distress Scale (K6), with disordered eating risk measured using EAT-8. Adjusted logistic regression models examined associations between food insecurity severity with psychological distress and disordered eating risk, controlling for age, living situation, and enrolment type. Mediation analysis explored whether psychological distress statistically mediated the association between food insecurity and disordered eating. **Results:** Overall, 63.2% of the 348 students surveyed reported some level of food insecurity, 15.8% met criteria for psychological distress and 42.0% were classified as being at high risk of disordered eating. In adjusted models, moderate (OR 2.46, 95% CI: 1.06–5.69) and severe food insecurity (OR 4.27, 95% CI: 1.83–9.97) were associated with higher odds of psychological distress. Severe food insecurity was also associated with higher odds of disordered eating risk (OR 2.07, 95% CI: 1.12–3.84). Mediation analysis indicated a statistically significant indirect association between food insecurity and disordered eating through psychological distress (B = 0.241, 95% CI: 0.065–0.418), with 43.5% of the total association statistically accounted for by psychological distress. **Conclusions:** Findings indicate an indirect statistical association in which food insecurity is associated with higher psychological distress, which is in turn associated with higher disordered eating risk, based on cross-sectional analysis. Longitudinal studies are needed to clarify temporality and better understand these relationships.

## 1. Introduction

Food insecurity is increasingly recognised as a major public health issue, defined as limited or uncertain access to sufficient, safe, nutritious, and socially acceptable food [1]. Food insecurity is a multidimensional construct, extending beyond food quantity to include access to nutritionally adequate diets and the ability to exercise choice over food consumption [1,2]. In high-income countries, food insecurity is shaped by a range of structural and social determinants, including income insecurity, rising living costs, housing stress, and limited access to affordable healthy food options [3]. Recent cost-of-living pressures have further heightened concerns about the affordability of food [4,5], particularly among population groups already experiencing financial precarity and structural disadvantage [6,7].

University students are increasingly identified as a group at heightened risk of food insecurity [8]. While tertiary education is often associated with future socioeconomic opportunity [9], the university period can also coincide with financial instability, insecure employment, and major life transitions [10]. Many students are managing tuition fees, housing costs, transport expenses, and reduced time available for paid work due to study commitments [11]. These pressures may be particularly pronounced among students living away from home [12], residing in shared accommodation [13], or studying as international students without access to established family and social support networks [14]. Food insecurity in this population has been associated not only with compromised food and nutrient intakes [15], but also with poorer academic performance [16] and poorer mental health outcomes [17,18]. Some international evidence suggests that students with food insecurity have 3–4 times higher odds of psychological distress and 2–3 times higher odds of poor self-rated mental health or depression, compared with food-secure peers [19,20], and these associations may be bidirectional [21]. The experience of uncertainty regarding food access, concerns about running out of food, and the need to compromise on food quality or quantity may contribute to chronic stress and emotional burden [22]. Food insecurity may also lead to feelings of deprivation, shame, and relative deprivation, leading to chronic stress [23]. Among university students, these experiences may intersect with academic stress and performance [24], social pressures [25], and broader financial hardship [26], potentially exacerbating mental health vulnerability.

Food insecurity has also been linked with disordered eating behaviours and increased risk of eating disorder symptomatology especially bulimic-spectrum disorders and binge eating in adults [27]. Among college students, food insecurity is associated with higher odds of disordered eating [28], and eating disorder diagnoses [29,30]. Periods of food scarcity or constrained food choice may contribute to irregular eating patterns, heightened preoccupation with food, restrictive eating, and episodes of loss-of-control or binge-type eating [31]. However, an important distinction remains regarding the extent to which these eating behaviours, particularly those arising following periods of restriction, reflect clinical eating disorders versus adaptive responses to constrained food access. Current diagnostic frameworks do not always explicitly account for food insecurity as a driver of eating behaviours, raising the possibility of misclassification. This highlights the need to interpret disordered eating risk within the broader context of food and nutrition security, particularly in populations experiencing material deprivation. Despite growing evidence of these associations, the pathways underpinning this relationship remain incompletely understood, warranting further research [32].

**Figure 1:**
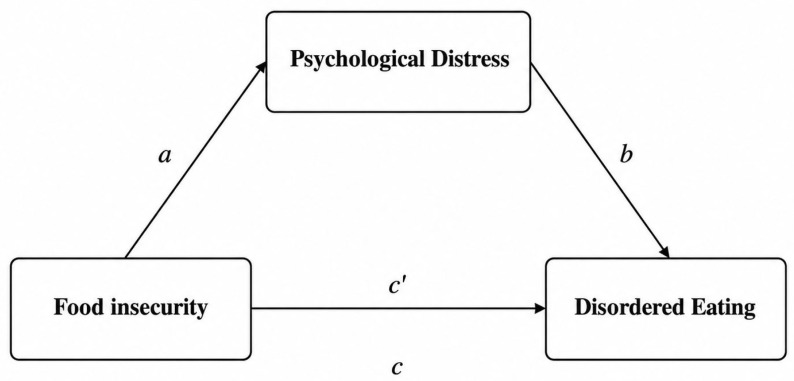
Conceptual framework of the relationship between food insecurity and disordered eating through psychological distress (mediator). Note. a = predictor to mediator path; b = mediator to outcome path; c′ = direct effect after adjustment for the mediator; c = total effect before adjustment for the mediator.

Psychological distress may statistically account for part of the association between food insecurity and disordered eating risk. Food insecurity is associated with poorer diet quality and nutrient intake, disrupting neurocognitive function and emotional regulation and worsening psychological distress [3,33]. It is also plausible that the stress associated with inadequate or uncertain food access contributes to poorer mental health, which in turn may be associated with adaptive eating behaviours that may arise as responses to stress and constrained food access, such as emotional eating, restrictive intake, or episodes of compensatory overeating. This proposed pathway is also supported by evidence from general population samples, where stress has been shown to explain a significant proportion of the association between food insecurity and eating disorder pathology, including binge eating–related appetitive traits such as emotional overeating and food responsiveness [34]. Different associations have been explored among student populations in relation to academic performance. For example, in UK students, food insecurity affected average grades indirectly via psychological distress, not directly [35]. In the USA, psychosocial health mediated most of the association between food insecurity and Grade Point Average [36]. Yet better understanding of whether psychological distress may help explain the association between food insecurity and disordered eating is needed to inform prevention and support strategies around food insecurity, mental health and eating disorders within university settings. Few studies have examined these relationships simultaneously among university students, particularly using mediation approaches to explore potential pathways. Therefore, the aim of this study was to examine the associations between food insecurity, psychological distress, and disordered eating risk among university students, and to explore whether psychological distress may mediate the association between food insecurity and disordered eating (Figure 1). It was hypothesised that greater food insecurity would be associated with higher psychological distress and higher disordered eating risk, and that psychological distress would statistically account for part of this association.

## 2. Materials and Methods

### 2.1. Study Design and Participants

A cross-sectional online survey was administered via Qualtrics (Qualtrics, Provo, UT, USA) to currently enrolled onshore University of Wollongong (UOW) students aged 18 years and over between May and July 2025. Convenience sampling approaches were used to encourage voluntary participation, including QR-coded posters placed around campus, flyers, and social media advertisements. Students were offered a free coffee voucher incentive redeemable at a university café upon completion of the survey. Ethics approval was granted by the UOW Human Research Ethics Committee (HREC2022/143).

### 2.2. Food Insecurity

Food security status was assessed using the validated six-item United States Department of Agriculture Household Food Security Survey Module (HFSSM; short form). This tool includes six items relating to food adequacy and affordability over the previous 12 months and has been widely used in student populations [4,8,37]. Responses were coded as affirmative or negative and summed to generate a total score ranging from 0 to 6. Food security status was categorised into four groups in line with previous research in Australian university students [4]: food secure (0), marginal food insecurity (1), moderate food insecurity (2–4), and severe food insecurity (5–6). For mediation analyses, food security status was additionally dichotomised as food secure (0) and food insecure (≥1, comprising marginal, moderate and severe food insecurity groups).

### 2.3. Psychological Distress

Psychological distress was assessed using the Kessler Psychological Distress Scale (K6) [6,38,39]. The K6 consists of six items assessing symptoms such as nervousness, hopelessness, restlessness, and sadness over the past 30 days. Responses were scored according to the Australian scoring system by ABS [7,39], producing a total score ranging from 6 to 30. For analysis, scores were dichotomised into no probable serious mental illness (6–18) and probable serious mental illness (19–30) [7].

### 2.4. Disordered Eating Risk

Disordered eating risk was assessed using the Eating Attitudes Test short form (EAT-8) [8,40]. The EAT-8 comprises eight items assessing symptoms such as guilt after eating, preoccupation with food, calories, body weight, and body shape. Responses of “never”, “rarely”, and “sometimes” were scored as 0, while “often”, “usually”, and “always” were scored as 1. Total scores ranged from 0 to 8. Consistent with validation studies, sex-specific cut-offs were applied, with participants classified as high risk if they scored ≥3 (females, non-binary) or ≥2 (males), and low risk otherwise [8].

### 2.5. Statistical Analysis

Data were exported into IBM SPSS Statistics (version 30; IBM Corp., Armonk, NY, USA) for cleaning and analysis. Responses with less than 50% completion were excluded. Descriptive statistics were used to summarise participant characteristics and prevalence estimates for food insecurity, psychological distress, and disordered eating risk.

Adjusted logistic regression models were used to examine the association between food insecurity severity (marginal, moderate, and severe compared with food secure) and (1) probable serious mental illness and (2) disordered eating risk. Models were adjusted for age group, living situation, and enrolment type (domestic or international student). Adjusted odds ratios (ORs) with 95% confidence intervals (CIs) were reported.

Mediation analysis was conducted to examine whether psychological distress statistically mediated the association between food insecurity and disordered eating. For this analysis, food insecurity was modelled as a binary predictor (food secure vs. food insecure), psychological distress as the mediator, and EAT-8 total score as the outcome. Psychological distress (K6) was treated as a continuous variable for mediation analysis. Covariates included age, living situation, and enrolment type (domestic vs. international). Mediation analysis was conducted using the PROCESS macro in SPSS v30 with 5000 bootstrap resamples to estimate indirect effects. Missing data were handled using listwise deletion. The EAT-8 total score was used as a continuous outcome to allow estimation of indirect effects, whereas the binary high-risk classification was used in logistic regression models. Direct, indirect, and total effects were estimated, and the proportion mediated was calculated. Statistical significance was determined using 95% confidence intervals and *p* values, with significance set at *p* < 0.05.

## 3. Results

### 3.1. Participant Characteristics and Key Study Outcomes

A total of 348 university students participated in the study (Table 1). Most participants were aged 18–24 years (75.9%), followed by 25–34 years (21.3%) and 35–44 years (2.9%). The majority identified as female (63.8%), while 34.2% were male and 2.0% identified as non-binary or third gender. Most participants were domestic students (63.2%), with 36.8% international students. Students were distributed across years of study, including first year (24.4%), second year (24.7%), third year (21.3%), fourth year (8.6%), and postgraduate study (21.0%). The most common living arrangement was rental or shared accommodation (42.8%), followed by living at home (30.7%) and on-campus student residences (25.0%).

Overall, 63.2% of students reported some level of food insecurity, including 15.4% marginal, 27.2% moderate, and 20.6% severe food insecurity (Table 1). Based on the K6 scale, 15.8% were classified as experiencing probable serious mental illness, with 42.0% classified as being at high risk of disordered eating according to the EAT-8.

### 3.2. Food Insecurity Severity and Psychological Distress

In adjusted logistic regression models controlling for age, living situation, and enrolment type, moderate and severe food insecurity were associated with higher odds of probable serious mental illness, compared with food-secure students (Table 2). Students experiencing moderate food insecurity had 2.46 times higher odds of probable serious mental illness (95% CI: 1.06–5.69), while those experiencing severe food insecurity had 4.27 times higher odds (95% CI: 1.83–9.97). Marginal food insecurity was not significantly associated with probable serious mental illness.

### 3.3. Food Insecurity Severity and Disordered Eating Risk

Table 3 presents adjusted models that examined the association between food insecurity severity and disordered eating risk. Severe food insecurity was associated with higher odds of disordered eating risk (95% CI: 1.12–3.84), while moderate food insecurity showed a borderline association (95% CI: 0.98–2.99). Marginal food insecurity was not associated with disordered eating risk.

### 3.4. Psychological Distress Adjusted Association and Mediation Analysis for Disordered Eating

**Figure 2:**
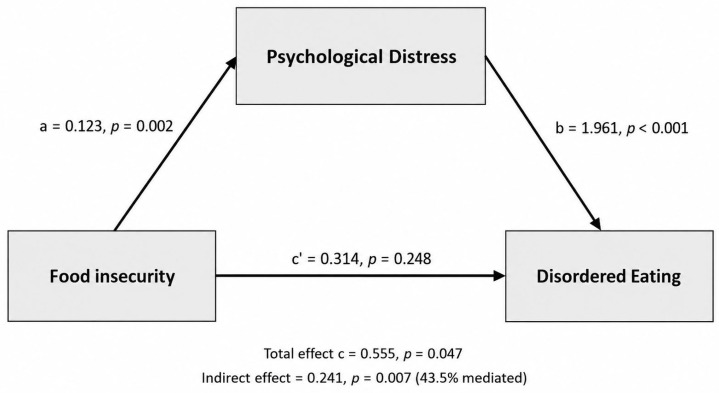
Mediation analysis of the association between food insecurity and disordered eating through psychological distress (cross-sectional statistical association).

Mediation analysis examined psychological distress as a mediator of the relationship between food insecurity and disordered eating (Table 4; Figure 2). Food insecurity was significantly associated with psychological distress (B = 0.123, 95% CI: 0.045–0.202, *p* = 0.002), and psychological distress was significantly associated with higher disordered eating scores (B = 1.961, 95% CI: 1.254–2.668, *p* < 0.001). The total effect of food insecurity on disordered eating was statistically significant (B = 0.555, 95% CI: 0.001–1.103, *p* = 0.047); however, the direct effect was no longer statistically significant after accounting for psychological distress (B = 0.314, 95% CI: −0.218–0.846, *p* = 0.248). The indirect effect of food insecurity on disordered eating through psychological distress was significant (B = 0.241, 95% CI: 0.065–0.418, *p* = 0.007), indicating that psychological distress mediated 43.5% of the total association.

## 4. Discussion

This study is among the first to examine the associations between food insecurity, psychological distress, and disordered eating risk among university students, and explore whether psychological distress may statistically account for the relationship between food insecurity and disordered eating. Several important findings emerged. First, food insecurity was highly prevalent in this sample, with almost two-thirds of students reporting some level of food insecurity. Second, increasing severity of food insecurity was associated with poorer mental health and higher disordered eating risk, with the strongest associations observed among students experiencing severe food insecurity. Third, mediation analysis suggested that psychological distress may represent an important association between food insecurity and disordered eating risk, accounting for nearly half of the total association. This pattern is statistically consistent with a possible pathway in which food insecurity is associated with psychological distress, which is in turn associated with disordered eating risk. However, given the cross-sectional design, the temporal direction of these relationships cannot be established within our study. Taken together, these findings suggest that psychological distress may statistically account for part of the association between food insecurity and disordered eating risk, reinforcing the need for integrated university responses that address both financial hardship and student mental health.

The high prevalence of food insecurity in this sample is concerning and reinforces growing evidence that university students are experiencing substantial material hardship. While this prevalence appears higher than estimates reported in the general Australian adult population (13%) [41], it is broadly consistent with studies among university students [8]. While emergency food relief initiatives, such as campus pantries and ad hoc food donations may provide important short-term support, these alone are unlikely to address the underlying drivers of student food insecurity [42]. Studies show many food insecure students do not use pantries, citing stigma, shame, perceived “insufficient need,” unsuitable food, poor information, and inconvenient hours [43]. These findings are consistent with broader calls for university responses that move ‘upstream’, beyond crisis response and towards more preventative and structural approaches that reduce financial and environmental barriers to adequate food access [44]. This may include social supermarkets on campus instead of food pantries, subsidised meal vouchers, low-cost healthy meals and free food initiatives linked to student engagement spaces [45]. While these supports are necessary, they should be considered short-term harm reduction strategies rather than standalone solutions. Positively, within Australia some universities such as the University of Tasmania [46] and University of New South Wales [47], have demonstrated sector leadership by implementing comprehensive food security strategies that extend beyond emergency food relief to include healthier campus food environments, food literacy initiatives, and broader structural supports.

A second key finding of this study was the severity gradient observed between food insecurity and psychological distress. Students experiencing moderate and severe food insecurity had substantially higher odds of probable serious mental illness, suggesting a severity gradient, whereby higher levels of food insecurity were associated with poorer mental wellbeing. This finding is consistent with research across other settings [48] suggesting that the relationship between worsening food insecurity and poorer mental health is not unique to the present setting. Within a university context, this has been shown to compound existing academic [16] and social stressors [49], creating a cumulative burden on mental health. Our findings add to the evidence that students presenting to campus health centres with psychological distress or disordered eating symptoms may also benefit from screening for food insecurity [50]. Similarly, students seeking support for food insecurity, such as through food pantries, may benefit from clear referral pathways to mental health, nutrition programs and dietetic services, warranting further investigation. Integrated models that recognise the intersection between material hardship, distress, and eating behaviours are likely to be more effective than siloed services [51].

The association between severe food insecurity and higher disordered eating risk observed in the current study also warrants careful consideration and provides, to our knowledge, the first Australian evidence among university students contributing to the growing body of literature suggesting that food insecurity is associated with an increased risk of eating disorder symptoms. This finding is consistent with international studies in university and community settings that have reported higher prevalence of binge eating, compensatory eating behaviours, and broader eating disorder pathology among individuals experiencing food insecurity [48]. Our mediation findings offer additional insight into how these relationships may be linked, at least in part, through psychological distress. While causality cannot be inferred from our cross-sectional design, these findings are consistent with the possibility that the emotional strain associated with food insecurity may be linked with eating behaviours that reflect adaptation to constrained food access. This may include emotional eating, reduced appetite regulation under stress, or cycles of restriction and compensatory eating [52]. Equally, it is plausible that disordered eating behaviours may exacerbate distress, or that these relationships are bidirectional [23]. However, future research that is longitudinal in design is needed to better understand the possible bi-directionality.

In light of these interrelated relationships, these findings, alongside the broader literature, suggest that effective responses should address the underlying drivers of food insecurity. Importantly, programs should be codesigned with students [53]. Yet, many university food security intervention programs are underfunded, fragmented, and unevaluated [54]. Evidence suggests that direct financial supports, including emergency hardship grants, subsidised accommodation, transport concessions, and targeted bursaries, may have greater impact than food relief alone because they address the underlying resource constraints driving food insecurity [55]. Financial assistance and income-based supports may be among the most effective interventions, as they restore agency and enable students to make their own food choices [56] in addition to policies to reduce the cost burden of higher education and housing [57,58]. Based on the findings of this study, solutions that combine financial and nutrition support with integrated mental health services may warrant consideration, and such services should be delivered in ways that are non-stigmatising and preserve student autonomy. Approaches that focus solely on emergency food provision without addressing dignity, choice, nutritional adequacy and mental wellbeing may be less effective and may inadvertently reinforce distress and poor mental health and wellbeing [56]. Future research is required to evaluate the effectiveness of these approaches on broad outcomes including food insecurity, psychological distress, academic outcomes within university settings.

A key strength of the current study is the use of validated measures of food insecurity, psychological distress, and disordered eating risk among this university student population. The study also moves beyond descriptive prevalence estimates by examining both adjusted associations and a mediation model, providing practical insight into how food insecurity may be linked with disordered eating risk through psychological distress, which adds important evidence to an emerging area of student health research. However, several limitations should be acknowledged. The cross-sectional design means that causality and temporal direction cannot be established. Accordingly, the mediation analysis should be interpreted as identifying a statistically significant indirect association, rather than demonstrating that psychological distress causally mediates the relationship between food insecurity and disordered eating risk. The study sample comprised students from a single institution using convenience recruitment, which may introduce selection bias and limit the representativeness of the findings. As such, the prevalence estimates and observed associations may not be directly generalisable to other Australian universities or to international student populations, where structural factors such as tuition costs, welfare supports, and campus food environments differ substantially. Further, the prevalence estimate should be interpreted cautiously given the convenience sampling strategy, voluntary participation, and use of a small food-related incentive. It is possible that students experiencing food insecurity, financial stress, psychological distress, or eating concerns were more motivated to participate, which may have inflated the prevalence estimates. Conversely, students experiencing severe hardship may also have been less able to participate due to time pressures, competing work or caring responsibilities, limited access to technology, or survey fatigue, meaning the magnitude and direction of selection bias cannot be determined with certainty. As such, the 63.2% prevalence observed in this study should not be interpreted as representative of all University of Wollongong students or Australian university students more broadly. Nonetheless, the estimate is broadly consistent with recent Australian university studies showing high and increasing levels of food insecurity among students. For example, Kent et al. (2025) [8] reported that food insecurity among Australian university students remained high across repeated cross-sectional surveys between 2022 and 2024, during a period of sustained inflation and cost-of-living pressure. While the mediation findings suggest that psychological distress may help explain the relationship between food insecurity and disordered eating, reverse or bidirectional relationships are also possible. The use of self-reported measures may introduce reporting bias, and the convenience sample from a single university limits generalisability to other student populations. Exposure, mediator, and outcome were measured over different timeframes (food insecurity: 12 months, psychological distress: 30 days, EAT-8: unspecified), which limits interpretation of temporal relationships. Both psychological distress and disordered eating scores in this study should be interpreted as indicators of risk rather than clinical diagnoses. The use of a ≥19 cut-off for the K6, aligned with Australian Bureau of Statistics guidance, may result in a more conservative estimate of psychological distress by capturing only more severe cases. This may underestimate the broader burden of moderate distress in student populations, although sensitivity analyses using a lower threshold (≥18) yielded comparable results, suggesting findings were robust to cut-off selection. Like most other studies, our analysis relied on global measures of perceived stress and did not differentiate between poverty-related stressors (e.g., financial strain, food access uncertainty) and other psychosocial stressors. This lack of specificity may obscure distinct mechanisms through which material deprivation influences eating behaviours. Further, while the EAT-8 is a validated screening tool, it does not distinguish between behaviours arising from clinical eating disorders and those occurring in the context of food insecurity or constrained food access. Importantly, some behaviours captured by the EAT-8—such as preoccupation with food or episodes of loss-of-control eating—may, in the context of food insecurity, reflect adaptive responses to periods of restriction rather than underlying eating disorder pathology. As such, the associations observed in this study should be interpreted as indicators of disordered eating risk rather than diagnostic of eating disorders. In addition, unmeasured factors such as prior mental health diagnosis, financial support, or existing eating disorder history may have influenced the observed associations. However, while selection bias may have influenced prevalence estimates, the observed graded associations between food insecurity severity, psychological distress and disordered eating risk remain important and warrant further investigation in larger, multi-institutional and representative samples.

## 5. Conclusions

Food insecurity is highly prevalent among university students and is associated with both psychological distress and disordered eating risk. The findings indicate that psychological distress may be an important pathway linking food insecurity and disordered eating, accounting for almost half of the observed association. These results support the need for direct, practical university responses that combine financial hardship support, affordable food access, nutrition support and integrated mental health services. Universities should treat food and nutrition insecurity as a core student wellbeing issue rather than a peripheral welfare concern. The most effective solutions are likely to be structural rather than crisis based, and should address affordability, food access, mental health and student autonomy.

## Figures and Tables

**Table 1:** Participant characteristics and key study outcomes.

	Characteristic	*n*	Valid %
Age group (*n* = 348)	18–24 years	264	75.9
	25–34 years	74	21.3
	35–44 years	10	2.9
Gender (*n* = 348)	Male	119	34.2
	Female	222	63.8
	Non-binary/third gender	7	2.0
Student status (*n* = 348)	Domestic student	220	63.2
	International student	128	36.8
Year of study (*n* = 348)	1st year (100 level)	85	24.4
	2nd year (200 level)	86	24.7
	3rd year (300 level)	74	21.3
	4th year (400 level)	30	8.6
	Postgraduate	73	21.0
Living situation (*n* = 348)	Own home or parents’ home	107	30.7
	On-campus student residence	87	25.0
	Renting or shared accommodation	149	42.8
	Other	5	1.4
Food security status (*n* = 345)	Food secure	127	36.8
	Marginal food insecurity	53	15.4
	Moderate food insecurity	94	27.2
	Severe food insecurity	71	20.6
Psychological distress (K6) (*n* = 348)	No probable serious mental illness	293	84.2
	Probable serious mental illness	55	15.8
Disordered eating risk (EAT-8) (*n* = 348)	Low risk	202	58.0
	High risk	146	42.0

**Table 2:** Association between food insecurity severity and psychological distress.

Variable	Adjusted OR *	SE	95% CI Lower	95% CI Upper	*p*
Marginal food insecurity	1.90	0.53	0.68	5.34	0.223
Moderate food insecurity	2.46	0.43	1.06	5.69	0.036
Severe food insecurity	4.27	0.43	1.83	9.97	<0.001

* Adjusting for age, living situation, and enrolment type (domestic/international).

**Table 3:** Association between food insecurity severity and disordered eating risk.

Variable	Adjusted OR *	SE	95% CI Lower	95% CI Upper	*p*
Marginal food insecurity	0.87	0.35	0.44	1.71	0.679
Moderate food insecurity	1.71	0.28	0.98	2.99	0.058
Severe food insecurity	2.07	0.31	1.12	3.84	0.020

* Adjusting for age, living situation, and enrolment type (domestic/international).

**Table 4:** Mediation analysis of the association between food insecurity and disordered eating through psychological distress.

Effect/Path	Description	β	SE	95% CI	*p* Value	% Mediated
a	Food insecurity → psychological distress	0.123	0.040	0.045, 0.202	0.002	–
b	Psychological distress → disordered eating	1.961	0.361	1.254, 2.668	<0.001	–
c′	Direct effect: food insecurity → disordered eating	0.314	0.272	−0.218, 0.846	0.248	56.5
Indirect (a × b)	Indirect effect through psychological distress	0.241	0.090	0.065, 0.418	0.007	43.5
Total (c)	Total effect	0.555	0.279	0.001, 1.103	0.047	100.0

Abbreviations: SE, standard error; CI, confidence interval. a = predictor to mediator path; b = mediator to outcome path; c′ = direct effect after adjustment for mediator; total (c) = total effect before adjustment. Indirect effect estimated using continuous EAT-8 score; associations are statistical and not causal.

## Data Availability

Data available upon written request to corresponding author due to ethical restrictions.

## Abbreviations

The following abbreviations are used in this manuscript:

ORs	Adjusted odds ratios
CIs	95% confidence intervals
SE	Standard Error
